# Pedestrian counting with grid-based binary sensors based on Monte Carlo method

**DOI:** 10.1186/2193-1801-3-299

**Published:** 2014-06-17

**Authors:** Shuto Fujii, Yoshiaki Taniguchi, Go Hasegawa, Morito Matsuoka

**Affiliations:** Graduate School of Information Science and Technology, Osaka University, 1-5 Yamadaoka, Suita-shi, 565-0871 Osaka, Japan; Faculty of Science and Engineering, Kindai University, 3-4-1 Kowakae, Higashi-osaka-shi, 577-8502 Osaka, Japan; Cybermedia Center, Osaka University, 1-32 Machikaneyama-cho, Toyonaka-shi, 560-0043 Osaka, Japan

**Keywords:** Pedestrian counting, Monte Carlo method, Binary sensor, Sensor network

## Abstract

**Abstract:**

In this paper, we propose a method for estimating the number of pedestrians walking in opposite directions, as in cases of a shopping street or a sidewalk in a downtown area. The proposed method utilizes a compound-eye sensor that is constructed by placing two binary sensors for the pedestrians’ movement direction and multiple binary sensors for the vertical direction of the pedestrians’ movement direction. A number of Monte Carlo simulations about the movement of pedestrians are conducted, and the output history of the compound-eye sensor is obtained in each simulation. The simulation scenario with a small difference of the output history of the compound-eye sensor is selected to estimate the number of pedestrians. Evaluation results show that in the field whose width is 8 [m] the relative error in the proposed method is the smallest by using 2×8 binary sensors.

## Introduction

In various fields such as marketing research, traffic control, and safety management, there is a demand for methods for estimating the number of pedestrians. For example, information on the temporal change in traffic volume of a footway can be used to determine the appropriate time for construction or maintenance (Leutzbach 1987). In addition, based on the number of pedestrians with information on their movement direction (i.e., the number of people entering and exiting) at the entrances of commercial facilities, event sites, or food courts, it is possible to prevent crowding. Although manual counting is often used for monitoring the number of pedestrians, it entails high labor cost and cannot be used in crowded environments. Therefore, automatic methods for estimating the number of pedestrians have attracted considerable attention (Dharmaraju et al. 2002; Bu et al. 2007; Greene-Roesel et al. 2008; Leykin and Hammoud 2006; Goubet et al. 2006; Hashimoto et al. 1998; Fod et al. 2002; Cui et al. 2007; Chen et al. 2008; Heeikkilä M and Pietikäinen 2006; Zhao and Wu 2008; Eshel and Moses 2008).

Most studies on the estimation of the number of pedestrians are conducted in the field of computer vision (Heeikkilä M and Pietikäinen 2006; Zhao and Wu 2008; Eshel and Moses 2008). By processing the video image obtained from a video camera, it is possible to estimate the movement characteristics of pedestrians, such as height, velocity, and pedestrian flow line, as well as the number of pedestrians. However, methods based on video processing require a strong resource base in terms of processing power, operating memory, storage, and electric power. In addition, the estimation accuracy of such methods is influenced by the brightness of the background.

On the other hand, a number of studies on pedestrian counting have used devices such as infrared imaging sensors (Leykin and Hammoud 2006; Goubet et al. 2006), passive infrared sensors (Hashimoto et al. 1998), laser sensors (Cui et al. 2007), ultrasonic sensors (Chen et al. 2008). In addition, there are commercial pedestrian counters using devices such as infrared imaging sensors (IRISYS people counter 2014), active infrared sensors (PCW-2BX03 directional people counter 2014), passive infrared sensors (Eco counter 2014), piezo films (Acoustic slab sensor 2014) and laser scanners (LOTraffic 2014). In particular, binary sensors, such as infrared sensors and piezo sensors, are among the simplest sensors, capable of detecting only the presence or absence of objects within the sensing region. Although binary sensors can neither detect the number of pedestrians nor identify individual pedestrians within the sensing region, they possess advantages such as low cost, simplicity, small size, and energy efficiency in comparison with other types of sensors. Therefore, methods for estimation of the number of pedestrians by using binary sensors have attracted attention. Since the capabilities of a single binary sensor are limited, as mentioned above, some researchers have considered using combinations of binary sensors for estimating the number of pedestrians together with their movement direction (Chen et al. 2008; Son et al. 2007; Lee 2009; Taniguchi and Nakano 2014). However, in these methods, the estimation accuracy significantly decreases in crowded environments where a large number of pedestrians move simultaneously.

In Fujii et al. (2013), we proposed a method for estimating the number of pedestrians and movement directions using a compound-eye sensor in environments where many pedestrians walk in opposite directions such as in a narrow corridors. The compound-eye sensor in this study is composed of ceiling-mounted passive binary sensors, such as pyroelectric infrared sensors (RE-210 2014), on a straight line. To estimate the number of pedestrians, we proposed a Monte Carlo-based method. The proposed method estimates the number of pedestrians based on the output history of the compound-eye sensor while the compound-eye sensor detects pedestrians and simulation results about the movement of virtual pedestrians. In Fujii et al. (2013), we showed that in crowed situations the proposed method is effective compared with an existing method. However, we assumed that the monitoring area is a narrow corridor where sensing region of a binary sensor covers width of a corridor. Since in wider streets, such as a shopping street, a sidewalk in a downtown area, the width of streets is more than 10 [m], the proposed method cannot be used in such monitoring areas.

**Figure 1:**
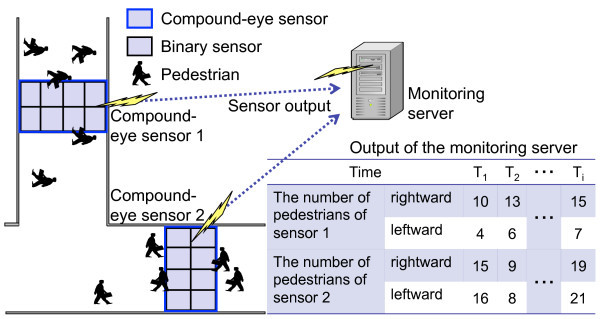
**Pedestrian counting system.**

In this paper, we propose a method for estimating the number of pedestrians in wider streets, such as a shopping street, a sidewalk in a downtown area, and so on. Figure 1 shows the system used for estimating the number of pedestrians in this paper. The compound-eye sensor in this paper is composed of mat-type binary sensors, such as piezo sensors (Measurement specialties Piezo Film Sensor 2014), on grid lines to deal with the pedestrian counting in wider streets. To estimate the number of pedestrians, we propose a Monte Carlo-based method similar to (Fujii et al. 2013). In the proposed method, a set of binary sensors that detect pedestrians locally is obtained from the output history of the compound-eye sensor. We call the set of binary sensors as a set of detecting binary sensors in this paper. Then, a number of simulations are conducted on the monitoring server for a set of detecting binary sensors. Finally, the monitoring server outputs the result in the most feasible simulation field with smaller difference between the output history of a set of detecting binary sensors and that of a set of virtual detecting binary sensors. We evaluate the relative error of the proposed method through simulation experiments by changing the specifications of binary sensor and the number of binary sensors.

The rest of this paper is organized as follows: the next section describes the pedestrian counting system and mobility model of pedestrians. The following section describes proposed pedestrian counting method in wide sidewalks and the next section describes simulation evaluations. The last section presents our conclusions and future works.

## Pedestrian counting system and mobility model of pedestrians

This section describes the pedestrian counting system, binary sensor models, the compound-eye sensor model used in the system, and a pedestrian mobility model.

### System overview

Figure 1 shows the pedestrian counting system, which consists of a monitoring server and sensor nodes. A sensor node consists of a wireless transceiver such as MICAz, IRIS Mote (IRIS Mote 2014) and Neo Mote (Neo Mote 2014) and multiple binary sensors, i.e. compound-eye sensor. A sensor node sends a data packet to the monitoring server when the output of its binary sensor changes. A data packet contains the sensor’s output value and a timestamp. The monitoring server uses the presence or absence of information from sensors for estimating the number of pedestrians walking in opposite directions. For simplicity, we assume that the system has a single sensor node, that data packets are reliably sent to the monitoring server, and that transmission latency is negligible.

In this section, the system is used for wide streets where a large number of pedestrians walk in two directions, such as in a local shopping street and a sidewalk in a downtown area. As the compound-eye sensor, we use mat-type binary sensors, such as piezo sensors (Measurement specialties Piezo Film Sensor 2014), on grid lines.

### Binary sensor model

**Figure 2:**
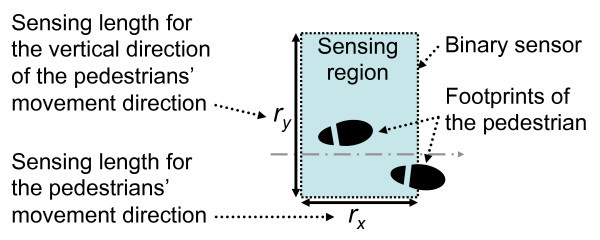
**Sensing model of a binary sensor.**

We assume a rectangular sensing region as shown in Figure 2. We refer to the distance of one side of the sensing region as the “sensing length.” We denote the sensing length for the pedestrians’ movement direction as *r*_*x*_ and the sensing length for the vertical direction of the pedestrians’ movement direction as *r*_*y*_. A binary sensor outputs a value of 1 when a foot of pedestrian steps on its sensing region and 0 when a foot of pedestrian steps away from its sensing region.

### Compound-eye sensor model

**Figure 3:**
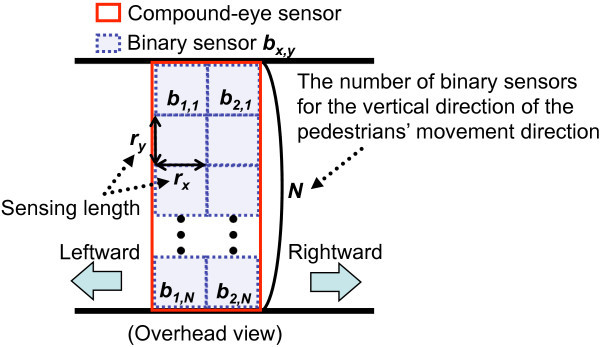
**Deployment of the compound-eye sensor.**

The compound-eye sensor consists of multiple binary sensors *b*_*x*,*y*_ (1≤*x*≤2,1≤*y*≤*N*) which are on grid lines by placing two binary sensors along the pedestrians’ movement direction, and *N* binary sensors along the vertical direction of the pedestrians’ movement direction as shown in Figure 3.

The region that is inside of sensing region of any binary sensor is denoted as the sensing region of the compound-eye sensor. The output of sensor *b*_*x*,*y*_ at time *t* is denoted as *o*_*x*,*y*,*t*_∈{0,1}. Furthermore, the output of the compound-eye sensor at time *t* is denoted as


When all outputs of binary sensors are 0, the number of pedestrians in the sensing region of the compound-eye sensor can be estimated as zero. We refer to this as an observable state. For other outputs the number of pedestrians cannot be determined, and this is referred to as an unobservable state. The interval from the moment when the state of the compound-eye sensor undergoes transition from an observable state to an unobservable state to the moment when the state undergoes transition to an observable state again is denoted as the unobservable interval.

### Mobility model of pedestrians

We need to decide location where a foot of a pedestrian steps, timing when a foot of a pedestrian steps on or steps away from ground since mat-type binary sensors are assumed. Therefore, we need a mobility model of pedestrians.

We first define the direction of moving from binary sensor *b*_1,*y*_ toward binary sensor *b*_2,*y*_ as right, and the opposite direction as left. We assume that pedestrians move either left (“leftward” pedestrians) or right (“rightward”) within the monitoring area, and they do not change their movement direction or velocity. The velocity distribution of pedestrians *v* is a normal distribution with mean *v*_*m*_ and deviation *v*_*σ*_.

**Figure 4:**
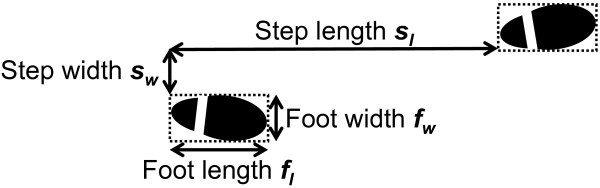
**Step length, step width, foot length, and foot width of pedestrians.**

The step length of pedestrians *s*_*l*_ follows a normal distribution with average *s*_*l*,*m*_ and deviation *s*_*l*,*σ*_. The step width *s*_*w*_, the foot length *f*_*l*_, and the foot width *f*_*w*_ of pedestrians are constant values since their variations are negligible compared to the variation of velocity of pedestrians *v* and that of step length of pedestrians *s*_*l*_. Figure 4 shows the step length, the step width, the foot length, and the foot width of pedestrians.

**Figure 5:**
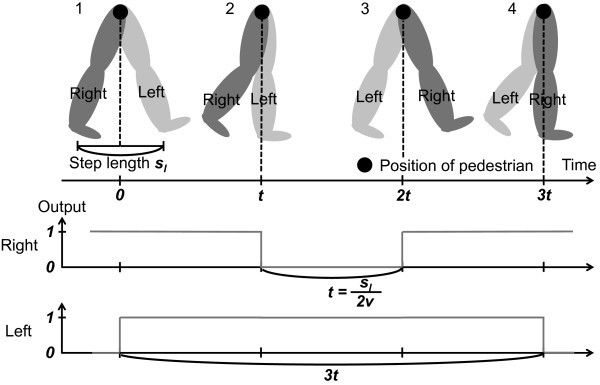
**Four states of walking motion and the timing of stepping on and stepping away from the ground of the foot of pedestrians (** Akutsu 1975 **).**

Next, we explain the timing of stepping on and stepping away from the ground. According to (Akutsu 1975), when we focus on one leg of pedestrian, the walking motion is classified four states as shown in Figure 5. For example, a pedestrian’s right leg steps away from the ground in the state 1, moves in the air in the state 2, steps on the ground in the state 3, and supports stepping away of a left leg in the state 4. In this paper, based on the walking motion model, the timing of stepping on and stepping away from the ground is defined as follows. The position of a pedestrian is defined as a position of groin. A back leg steps away from the ground when the position of a pedestrian reaches to the distance of the step length from the position of the back leg. An anterior leg steps on the ground when the position of the pedestrian reaches to half distance of the step length from the position of the back leg.

## Pedestrian counting method

This section describes the proposed pedestrian counting method based on Monte Carlo method for estimating the number of pedestrians in wide sidewalks. The proposed method consists of two sub-methods, one for determining a set of binary sensors that detects pedestrians, and one for estimating the number of pedestrians in the set of binary sensors by conducting Monte Carlo simulations.

### Overview

A part of adjacent binary sensors in the compound-eye sensor detect pedestrians when the compound-eye sensor is in an unobservable interval since the compound-eye sensor consists of multiple binary sensors on grid lines. In this paper, the adjacent binary sensors that detect pedestrians are referred to as detecting binary sensors.

The proposed method first determines a set of detecting binary sensors by memorizing binary sensors that detect pedestrians during the compound-eye sensor is in an unobservable interval. Secondly, the proposed method estimates the number of pedestrians by conducting a number of Monte Carlo simulations. In particular, the monitoring server generates a simulation field with a set of virtual detecting binary sensors, randomly generates virtual pedestrians, moves them in the simulation field, and updates the output history of a set of virtual detecting binary sensors. Simulations are continued until a fixed number of simulation results are obtained which have a smaller difference between the output history of a set of detecting binary sensors and that of a set of virtual detecting binary sensors. Finally, the proposed method outputs the number of pedestrians by selecting the most feasible simulation result. In the following sections, we explain a sub-method for determining a set of detecting binary sensors, and describes a sub-method for estimating the number of pedestrians with a set of detecting binary sensors.

### Determining a set of detecting binary sensors

**Figure 6:**
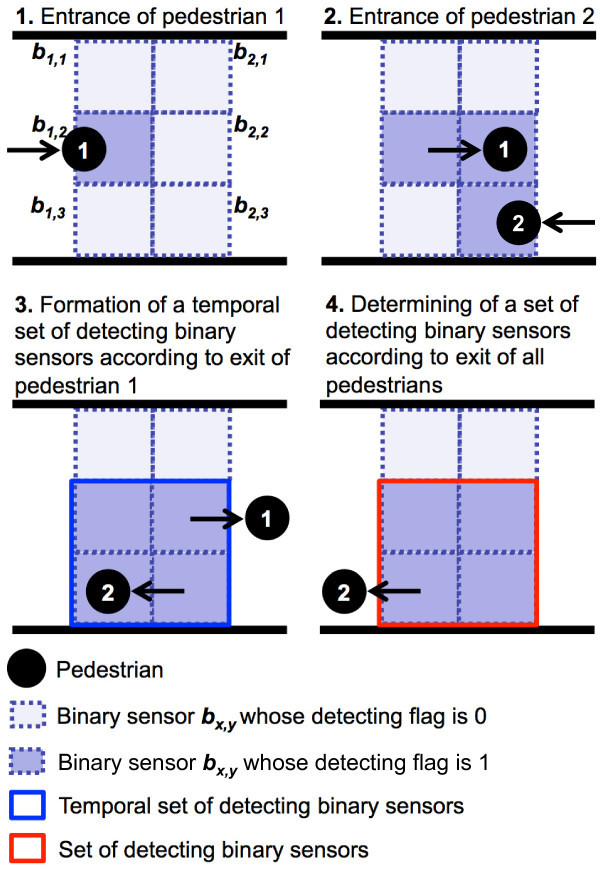
**Example of a set of detecting binary sensors.**

First, we explain the process for determining a set of detecting binary sensors. An example for determining a set of detecting binary senors is shown in Figure 6. In the proposed method, a flag is maintained for each binary sensor to maintain whether a binary sensor detected pedestrians or not. The flag is referred to as a detecting flag. The output of a binary sensor indicates whether pedestrians are in the sensing region of its binary sensor or not. On the other hand, the detecting flag of a binary sensor indicates whether pedestrians were in the sensing region of its binary sensor before or not. A detecting flag of each binary sensor is initially set to 0. When a binary sensor detects pedestrians, the detecting flag of the binary sensor is set to 1.

When output of a rightmost or leftmost binary sensor of the compound-eye sensor changes from 1 to 0, all pedestrians may exit the sensing region of a set of detecting binary sensors. Therefore, the proposed method checks whether a set of detecting binary sensors is determined or not as follows.

1. When the output of a right (left) binary sensor *b*_1,*y*_ (*b*_2,*y*_) (1≤*y*≤*N*) of the compound-eye sensor changes from 1 to 0, a temporal set of detecting binary sensors  is initialized as . After that, as long as there is adjacent binary sensor *b*_*x*′,*y*′_ whose detecting flag is 1, the temporal set of detecting binary sensors  is updated as .
2. If all outputs of binary sensors in the temporal set of detecting binary sensors  are 0, that is, if there is no pedestrian in the sensing region of , the temporal set of detecting binary sensors  is considered as a set of detecting binary sensors . After that, all detecting flags of binary sensors in the set of detecting binary sensors  are set to 0. Otherwise, the temporal set of detecting binary sensors  is deleted.

### Pedestrian counting in a set of detecting binary sensors

Next, we explain the process for estimating the number of pedestrians in a set of detecting binary sensors  in an unobservable interval. The proposed method maintains a table to memorize a fixed number of simulation results (hereinafter, simulation results table) whose size is *X*. The simulation results table maintains the difference of the output history *Δ*_*k*,*j*_, the number of virtual rightward pedestrians , and the number of virtual leftward pedestrians  in simulation results *s*_*k*,*j*_ (1≤*j*≤*X*). The difference of the output history  in the simulation results table is initialized to a large value.

Now, a set of detecting binary sensors  is assumed to be composed of 2×*n* binary sensors (1≤*n*≤*N*). The time when the first pedestrian enters into the sensing region of the set of detecting binary sensors  is denoted as *t*_0_. Outputs of binary sensors in the set of detecting binary sensors  is assumed to change *L* times in the unobservable interval of the set of detecting binary sensors . The time when the output of the set of detecting binary sensors  changes *i*-th times (0≤*i*≤*L*) is denoted as *t*_*i*_. The output of the set of detecting binary sensors  at time *t*_*i*_ is denoted as


**Figure 7:**
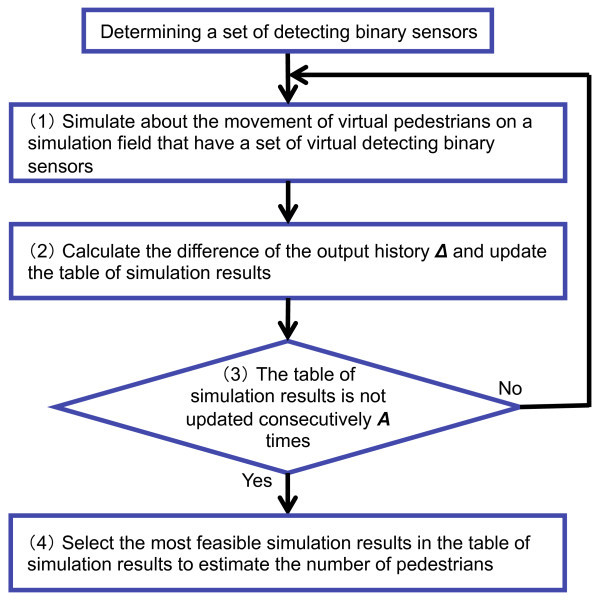
**Flowchart of the sub-method for estimating the number of pedestrians using a set of detecting binary sensors.**

The output history of the set of detecting binary sensors  is denoted as .Figure 7 shows the flowchart of the sub-method for estimating the number of pedestrians in a set of detecting binary sensors. The sub-method acts as follows:

1. When the set of detecting binary sensors  is determined, the monitoring server begins estimating the number of pedestrians for the set of detecting binary sensors . The monitoring server first generates a simulation field which has a set of virtual detecting binary sensors  composed of 2×*n* virtual binary sensors. Then, in the interval *t*_*L*_-*t*_0_, the monitoring server generates virtual pedestrians and moves them based on statistical information on pedestrians such as arrival rate, velocity, step length, and so on. These information are assumed to be obtained preliminary by measuring in the monitoring field. For a simulation field, the monitoring server maintains the number of virtual rightward and leftward pedestrians entered into the sensing region of the set of detecting binary sensors. It also maintains the output history  of the set of virtual detecting binary sensors. The time when the first pedestrian enters into the sensing region of the set of virtual detecting binary sensors  is denoted as *t*0′=*t*_0_. Outputs of binary sensors in the set of virtual detecting binary sensors  is assumed to change *L*^′^ times until the time *t*_*L*_. The time when the output of the set of virtual detecting binary sensors  changes *i*^′^-th times (0≤*i*^′^≤*L*^′^) is denoted as *ti*^′^′. The output of the virtual binary sensor *bx*,*y*′ at time *ti*^′^′ is denoted as  and the output of the set of virtual detecting binary sensors  at time *ti*^′^′ is denoted as . The output history of the set of virtual detecting binary sensors  in the simulation field is denoted as .
2. The monitoring server calculates the difference  between the output history of the set of detecting binary sensors  and that of the set of virtual detecting binary sensors  in the simulation field as follows:
1
  where *δ*(*T*_*i*_) is next equation:
2
  *T*_*i*_ is the *i*-th time (0≤*i*≤*L*+*L*^′^-1) of a union set of times {*t*_0_,*t*_1_,…,*t*_*L*_}∪{*t*0′,*t*1′,…,*tL*^′^′} in order of time. Equation (2) indicates the sum of the Hamming distance between the output of the binary sensor *b*_*x*,*y*_ and that of the corresponding virtual binary sensors *bx*,*y*′ for all binary sensors in the set of detecting binary sensors  at time *T*_*i*_. Equation (1) indicates the sum of the value of output time weighting of Equation (2). When the difference of the output history  is smaller than the biggest difference of the output history  in the simulation results table, the *j*_*max*_-th entry in the simulation results table is replaced to the new entry: the difference of the output history , the number of virtual rightward pedestrians , and the number of virtual leftward pedestrians .
3. If the simulation results table is not updated consecutively *A* times, the sub-method moves to step 4. Otherwise it moves to step 1. We refer to *A* as the simulation termination threshold.
4. The monitoring server calculates the median value of the difference *Δ*_*k*,*mid*_ from the simulation results table. Then, it selects a set of feasible simulation results  as follows:
3
  Next, the monitoring server selects the most feasible simulation results *s*_*k*,*f*_ with the median value in terms of the total number of virtual pedestrians  from a set of feasible simulation results . After that, the monitoring server chooses  as the estimated number of rightward pedestrians  and  as the estimated number of leftward pedestrians .

## Performance evaluation

This section evaluates the accuracy of the proposed method through simulation experiments.

### Simulation settings

We evaluate the proposed method through simulations. To evaluate the basic characteristics of the proposed method, we set the mean and deviation of the velocity of pedestrians *v*_*m*_, *v*_*σ*_, the mean and deviation of the step length of pedestrians *s*_*l*,*m*_, *s*_*l*,*σ*_ according to Akutsu (1975). We assume that the monitoring server knows the step width of pedestrians *s*_*w*_=12 [cm], the foot length *f*_*l*_=25 [cm], and the foot width *f*_*w*_=10 [cm], respectively (Akutsu 1975). We also assume that the width of fields is 8 [m], the leftward pedestrian arrival rate and the rightward pedestrian arrival rate are the same, in other words, *λ*_*l*_=*λ*_*r*_=*λ*. Leftward (rightward) pedestrians arrive from the right (left) side of the monitoring area by a Poisson process with an arrival rate *λ*.

To evaluate the estimation accuracy of the proposed method, we use relative error *e* as an evaluation index:
4

Here, a smaller relative error indicates higher estimation accuracy.

### Basic characteristics

We first evaluate the basic characteristics of the proposed method by changing the size of the simulation results table *X* and the simulation termination threshold *A*. In this evaluation, we use an unobservable interval of a set of detecting binary sensors as one evaluation interval since we want to evaluate the relative error and the average number of simulation trials of each set of detecting binary sensors. We assume that the monitoring server knows the arrival rate of pedestrians *λ*. Table 1 shows other parameters of the first performance evaluation.

**Figure 8:**
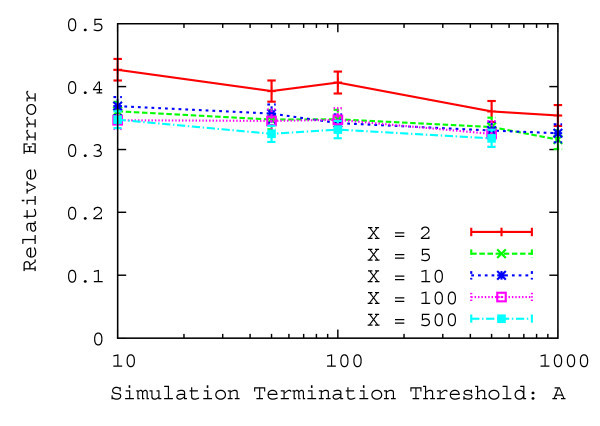
**Relative error as a function of the simulation termination threshold.**

**Figure 9:**
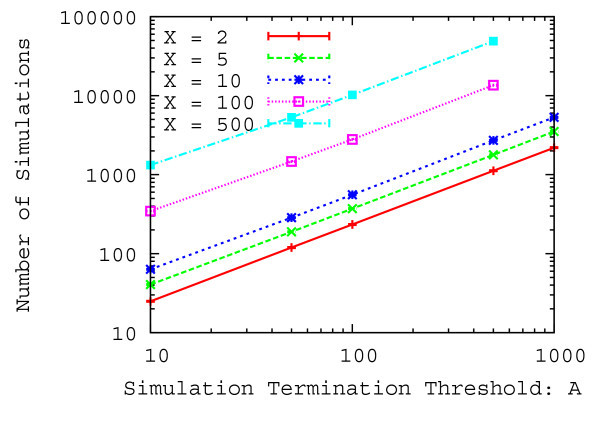
**Average number of simulation trials as a function of the simulation termination threshold.**

Figures 8 and 9 show the relative error and the average number of simulation trials with a 95% confidence interval as a function of the size of the simulation results table and the simulation termination threshold over 2000 evaluations.

As shown in Figure 8, the relative error decreases when the size of simulation results table increases from 2 to 5. However, the relative error does not decrease when the size of simulation results table increases from 5. In addition, the relative error slightly decreases when the simulation termination threshold increases. As shown in Figure 9, the number of simulation trials increases considerably when the size of simulation results table and the simulation termination threshold increases. In particular, it is noticeable when the size of simulation results table is large. Therefore, the relative error decreases when the number of simulation trials increases, however, the relative error does not decrease any more even if several tens of thousands of simulations are tried. This is because simulation results that have smaller difference of the output history are obtained within ten thousand simulation trials. Hence, in what follows, the size of simulation results table is set to 5 and the simulation termination threshold is set to 1000 since the number of simulation trials is small and the relative error is also small.

**Table 1:** **Parameters of the performance evaluation as a function of the size of the simulation results table and simulation termination threshold**

Parameters	Values
Size of the simulation results table *X*	2, 5, 10, 100, 500
Simulation termination threshold *A*	10, 50, 100, 500, 1000
Number of binary sensors 2×*N*	2 × 8
Sensing length *r*_*x*_ [m]	0.9
Sensing length *r*_*y*_ [m]	1.0
Arrival rate *λ* [people/sec]	0.5

**Figure 10:**
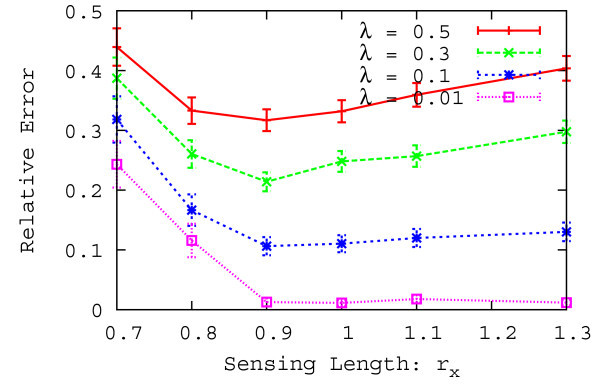
**Relative error as a function of the sensing length.**

We next evaluate the basic characteristics of the proposed method by changing the sensing length *r*_*x*_ and the arrival rate of pedestrians *λ*. In what follows, we use an unobservable interval of the compound-eye sensor as one evaluation interval. Table 2 shows other parameters of the second performance evaluation. Figure 10 shows the relative error with a 95% confidence interval as a function of the sensing length over 1000 evaluations.

As shown in Figure 10, the relative error increases when the arrival rate becomes large because the unobservable interval increases with arrival rate. In addition, when the sensing length becomes large, the relative error transiently decreases and then increases. When the sensing length is small, the relative error increases because the sensing length decreases in size compare to the step length. In this case, a leftward (rightward) pedestrian has a potential not to step on the sensing region of right (left) binary sensor and to step on the sensing region of left (right) binary sensor, so the relative error increases. When the sensing length is large, the sensing region becomes large and the number of pedestrians increases in an unobservable interval, thus, the relative error increases.

**Table 2:** **Parameters of the performance evaluation as a function of the sensing length and the arrival rate of pedestrians**

Parameters	Values
Size of the simulation results table *X*	5
Simulation termination threshold *A*	1000
Number of binary sensors 2×*N*	2 × 8
Sensing length *r*_*x*_ [m]	0.7, 0.8, 0.9, 1.0, 1.1, 1.3
Sensing length *r*_*y*_ [m]	1.0
Arrival rate *λ* [people/sec]	0.01, 0.1, 0.3, 0.5

### Accuracy as a function of the number of binary sensors

**Figure 11:**
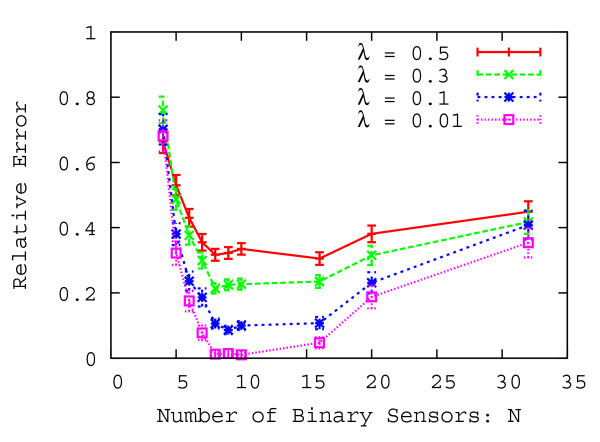
**Relative error as a function of the number of binary sensors.**

We finally evaluate the characteristics of the proposed method by changing the number of binary sensors *N*. Table 3 shows other parameters of the performance evaluation. Figure 11 shows the relative error with a 95% confidence interval as a function of the number of binary sensors over 1000 evaluations.

**Table 3:** **Parameters of the performance evaluation as a function of the number of binary sensors**

Parameters	Values
Size of the simulation results table *X*	5
Simulation termination threshold *A*	1000
Number of binary sensors 2×*N*	2 × 4∼10, 16, 20, 32
Sensing length *r*_*x*_ [m]	0.9
Sensing length *r*_*y*_ [m]	2.0 ∼0.25
Arrival rate *λ* [people/sec]	0.01, 0.1, 0.3, 0.5

As shown in Figure 11, when the number of binary sensors increases, the relative error transiently decreases and then increases. When the number of binary sensors increases and multiple pedestrians enter into the sensing region of the compound-eye sensor, multiple sets of detecting binary sensors are more likely to detect pedestrians apart. Therefore, the number of pedestrians decreases in each set of detecting binary sensors and the relative error for the number of estimated pedestrians in the compound-eye sensor decreases. However, the relative error increases when the number of binary sensors increases too many. This is because the sensing length *r*_*y*_ becomes smaller with increasing the number of binary sensors. When the sensing length becomes small, a pedestrian is more likely to step on multiple binary sensors and the output history of the compound-eye sensor becomes complex, so the relative error increases.

## Conclusions and future works

In this paper, we proposed a Monte Carlo-based method for estimating the number of pedestrians moving in opposite directions using binary sensors in wide streets. Simulation results show that the relative error is the smallest by using 2×8 binary sensors when a width of fields is 8 [m].

In future works, we plan to evaluate the performance of the proposed method in real environments through implementation and experiments. We also plan to extend the proposed method to handle multiple movement directions.
